# Prolapse of the Iris

**Published:** 1876-12

**Authors:** T. M. McIntosh

**Affiliations:** Atlanta, Ga.


### PROLAPSE OF THE IRIS.

By T. M. McINTOSH, M.D., Atlanta, Ga.

*(Read before the Atlanta Academy of Medicine.)*

I propose to give, in an imperfect way, the definition, cause, results, and treatment of prolapse of the iris, reporting illustrative cases.

A prolapse of the iris is a protrusion of a portion of its substance through an opening in the cornea or sclerotic, which is produced by the knife in the operation for cataract by extraction, by perforating ulcers of the cornea, and by traumatic injuries.

A prolapse may be of any degree, from a mere spec projecting through the opening, to quite an extensive protrusion, producing great displacement, and even obliteration, of the pupil; and where the opening is in the centre of the cornea, a partial or complete destruction of the anterior chamber, the iris being adherent to the posterior portion of the cornea.

Because of the deformity, and in some cases the inflammation and remote danger to the eye which a prolapse of the iris occasions, it should always be watched with care, and judiciously treated. As after the operation for cataract by extraction it frequently occurs, and as the treatment in those cases will give a general idea of the method to be pursued where it occurs from other causes, except when following ulcers of the cornea, where the only difference in the indications to be met will be dependent upon the character of the ulceration, I shall give the treatment pursued in these cases occurring after the extraction of the cataract.

After such an operation all may go on well for a day or so, when, if a prolapse occur, it will be found that, without any apparent cause, or immediately following a cough, a sneeze, or or some untoward movement of the head, the eye began to run water; that the patient was restless and disturbed, slept very little during the night, or maybe was a little feverish, with a feeling as if grit or sand was in the eye. Upon examining the dressing it is found to be more than usually soiled, and upon it there may be a small amount of pus. If the symptoms of irritation be great, as increase of lachrymation, a flow of aqueous humor, ciliary neuralgia, with other symptoms of commencing or developed iritis, the upper lid should be carefully raised and the eye examined to ascertain the condition of the structure within, when a prolapse of the iris will be in most instances found, sometimes a very small one, or so large as to be seen on the most careless inspection.

If much of the iris be without the opening, and the section gape much, it may lay the foundation for the occurrence of a staphyloma, of both the cornea and the iris, in the future. But this does not usually occur, for it has been known that the iris filled the entire section after the operation for catarct by the flap method; the section so widely gaping that it finally contracted and disappeared, the edges of the incision growing together, leaving the cornea of the proper curvature, and with as good vision as is usually obtained after cataract operations; or the prolapsed iris, subject to the irritating influences of the air, foreign substances, and the friction of the lids, may light up an acute iritis that may be of immediate danger to the eye, or result in a low form of irido-choroditis, which causes but slight pain, but little deposits of lymph, and but little cloudiness of the aqueous humor, but which is a very serious disease. By its masked form it is apt to go unnoticed until serious changes have taken place in the tissues within the globe, when the failing vision directs attention toward it, though in this class of cases, even if recognized early, treatment is very unsatisfactory, nothing being of much benefit except an iridectomy, repeated if need be; this in many cases doing but little to arrest the progress of the disease, which may finally end in atrophy of the globe. Fortunately, however, the majority of cases do not pursue a course so unfavorable, and an extensive prolapse will often dwindle away, leaving most excellent vision, without the least symptoms occurring during the existence of the prolapse up to the period of its disappearance, that would lead any one to suspect such an occurrence.

The prolapse resulting from a perforating ulcer is merely an exaggeration of nature's effort to repair the breach, for when perforation is complete, the aqueous humor being evacuated, the iris falls forward in the opening, and adhesions take place between it and the edges of the ulcer, close it and prevent the constant flow of aqueous humor through the fistula, keeping the tissue at rest, and thereby allowing the regeneration of the corneal tissue. After this, the opening being closed, the aqueous humor reaccumulates, the dilating action of the longitudinal fibres of the iris comes into play, and tears the adhesions that had formed, and the iris assumes the normal position; or the adhesions being too firm to be thus torn, are permanently attached to the cornea, leaving a shallow anterior chamber and displaced pupil.

The Traumatic prolapsus is caused by bits of steel, stone or other substances coming with force against the ball and penetrating, and when the aqueous humor gushes out it brings a portion of the iris. This injury is usually followed by severe inflammation, as often there is considerable concussion of the eye produced by the blow upon it.

The first case that I will report followed the extraction of a cataract in a woman seventy-one years old. I made no iridectomy for the reason that the attachments of the lens, being much weakened by age, during the disturbance of the eye necessary for the different steps of the operation, were broken loose, so that when the aqueous humor flowed out upon the section being completed, bringing a portion of the iris. The lens followed to some extent and presented in a favorable position to be removed, which was done in the usual way. I then replaced the iris and bandaged the eye. Everything went on well, nothing wrong being suspected, but at the end of six days, when I opened the eye, I found to my surprise a considerable prolapse of the iris, distended with aqueous humor. As no unfavorable symptoms had thus far shown themselves I concluded to leave it alone and watch it closely and upon the first appearance of any unfavorable sign to puncture the vesicle and clip off the prolapsed iris with the scissors, as is the advised method.

I only used a light bandage to fix the eye and atropine as a local sedative and to lessen the tendency to inflammation, and watched day by day the prolapse dwindle, grow less and completely disappear, the edges of the incision growing together, leaving a pupil of the natural size, somewhat responsive to light and a little displaced; and since, there has never been one particle of pain in the eye; vision as good as before blindness.

Second case: 68 years of age—operation, cataract; no iridectomy for the same reason as in first case. On the third day after the operation, for a few hours, there was some little gritty feeling about the eye, which I presumed was occasioned by the occurrence of a prolapse, which, three days later, upon opening the eye I found to be the case. Having so good a result in the first case with only the bandage and atropine, I concluded to try the same again, only using some slight pressure with the bandage, and was rewarded with equally as good results. Pupil a little displaced upward. Vision excellent.

Third case, a man 63 years old. Operation, cataract, iridectomy. As his pupil did not dilate freely under the influence of atropine previous to the operation, I feared there might be some inflammation, and so stated to the patient. On the morning of the third day after operation, upon removing the dressing I found it, damper than usual with a little pus upon it, and the patient complaining of grittiness and soreness of the eye and restlessness the night before. These symptoms I refered to a protrusion of the iris, but as they did not increase I did not open the eye until the fifth day after the operation, when I found considerable redness of the conjunctiva and some iritis—more than the symptoms would have indicated—and a prolapse of the iris from one angle of the incision. I used the bandage with pressure and atropine and a mild astringent for the conjunctivitis, which subsided to a great extent, but the prolapsus did not disappear very rapidly, nor did the iritis subside but little. Thinking that the prolapse kept up these troubles, I snipped it off with scissors, which was followed by considerable reaction, aggravating the symptoms and retarding the cure more, in my opinion, than if I had left the prolapse to itself. Vision: reads no. xx snellen with tolerable facility.

There is nothing specially interesting in these cases except the entire absence of any symptoms of irritation in two of them during the existence of the prolapse and since, and the simplicity of treatment adopted, which is contrary to the usually advised method according to recent works, which lay stress upon the danger to the eye that a prolapse will occasion, and the immediate snipping off, because of immediate inflammation; or the constant teasing to which the iris is subject in consequence of the inability to dilate under the influence of light, leading to a chronic inflammation that will destroy the vision.

In one of the cases, it is true, there occured some inflammation, but whether the result of the prolapse I am not satisfied, for the iris was not healthy previous to the operation, and the inflammation would have been likely to have occured any way; the prolapse may have aggravated it some, but clipping it off intensified the trouble.

Certainly every case of prolapse of the iris may not prove so harmless as these, and I do not by any means think that, though faequently they are devoid of the least real danger, that the surgeon should not use every care to prevent such an occurrence.

